# A Simple Minimum-Setup Pipeline for Using Leg-Worn Inertial Sensors to Track Knee Flexion: Validation on 10 Movements

**DOI:** 10.3390/s26123704

**Published:** 2026-06-10

**Authors:** Ke Song, Josh R. Baxter

**Affiliations:** Department of Orthopaedic Surgery, University of Pennsylvania, Philadelphia, PA 19104, USA; josh.baxter@pennmedicine.upenn.edu

**Keywords:** wearable sensors, inertial measurement unit, motion analysis, kinematics, joint angle, lower limb

## Abstract

**Highlights:**

**What are the main findings?**

- We established a simple pipeline with minimum experimental requirements for leg-worn inertial measurement unit (IMU) sensors to estimate the knee flexion angle;
- IMU-estimated knee flexion demonstrated good agreements with optical motion capture (both marker-based and markerless) during most daily living activities.

**What are the implications of the main findings?**

- Our validated simple pipeline makes IMU-based knee motion tracking more practical and compatible with clinical research;
- Future research should seek best practices on sensor wearing to secure valid continuous data in clinically relevant environments.

**Abstract:**

Knee motion is a key biomarker in chronic musculoskeletal diseases, yet conventional in-lab optical motion capture falls short of identifying how knee motion continuously impacts joint health outside the lab. Inertial measurement unit (IMU) provides a clinically attractive approach for continuous real-world motion tracking. Our goal was to establish a clinically practical, minimum-setup pipeline for leg-worn IMUs to estimate knee flexion and determine its concurrent validity to optical motion capture during various knee movements. We recorded thigh and shank-worn IMU data with concurrent marker-based and markerless optical motion capture on 10 healthy adults, who performed 10 common movements including walking, running, and stair navigation. We combined IMU functional alignment with data fusion to estimate knee flexion during each movement and compared IMU-based estimate against both motion capture systems using Pearson correlation (R_xy_) and root-mean-square difference (RMSD). IMU-estimated knee flexion strongly correlated with motion capture (R_xy_ ≥ 0.9). RMSDs were smaller for slower movements like walking (RMSD = 4.4–6.0°) while larger during faster movements like running (RMSD = 5.4–9.4°). Wearable IMUs track knee flexion with comparable results to motion capture during daily activities typical to older adults, highlighting their potential for continuous patient monitoring. Our simple pipeline makes IMU-based knee motion tracking more practical and compatible with clinical research. Future research should seek IMU-wearing best practices to secure clinically meaningful data on real-world knee mobility.

## 1. Introduction

Knee motion is an established biomarker in chronic musculoskeletal diseases like knee osteoarthritis [1,2] and athletic overuse conditions like patellofemoral pain and tendinopathy [3,4]. Clinical gait analyses found that knee osteoarthritis reduces knee flexion during walking [1,2]. A recent study found that patients whose osteoarthritis progressed in 3 years had smaller early-stance knee flexion at baseline than those who did not progress [5], indicating a causal impact. Despite its clinical importance, knee motion analysis in clinical assessments have mostly relied on marker-based motion capture. Clinical motion analysis often only includes walking or few selected exercises, possibly limited by the time and effort required for tedious marker placement and labeling. Although markerless optical motion capture [6,7,8] has improved suitability for clinical use, it demands computation time and data management [9], is still constrained to a fixed capture space, and cannot track motion over long durations (e.g., days). Isolated in-lab analysis also does not capture the large variability of knee motion during daily living activities or rehabilitation exercises [10], nor the cumulative pattern of abnormal motion. These limitations hinder our ability to determine the roles of real-world knee function in knee pathology and clinical treatments.

Inertial measurement unit (IMU) is an increasingly popular and clinically attractive approach towards fully portable and continuous real-world motion capture [11,12]. IMU worn on a body segment can track its orientation via data fusion, by integrating gyroscope-based angular rate for segment angle and using accelerometer-measured gravity to correct for drift caused by gyroscope noise [13,14]. Researchers have leveraged this working principle to track thigh and shank orientations in the sagittal plane and resolve the knee flexion angle, which expands our ability to quantify knee mobility outside of traditional laboratory and clinic settings [11,12]. While many studies have compared IMU-estimated knee flexion to marker-based [15], approaches and results have been highly variable. Few have compared both IMU and markerless optical motion capture concurrently to a marked-based system; we are only aware of one such study, limited to four athletic activities [9]. No study has compared IMU directly to markerless optical motion capture, or across a large variety (10+) of clinically relevant knee movements on a single cohort. Concurrent multi-system comparisons across slow (e.g., gait, daily living functions) and fast activities (e.g., running, sport exercises) will draw valuable insights into population-specific pros and cons of IMU motion tracking versus both field standards (marker-based) and emerging technologies (markerless optical). These insights will help researchers, clinicians, and athletic trainers select the most suitable motion tracker. Benchmarking IMU-based kinematics against optical motion capture will also support multiple-site translational studies and integrating in-lab with real-world assessments.

Importantly, while many innovative studies have advanced the technical frontier of IMU human motion tracking [15,16,17,18,19], much less focus has been placed on translating state-of-the-art algorithms toward clinically practical paradigms. Almost all biomechanical IMU studies cite clinical or real-world applications as primary motivation. Yet in our experience, clinical researchers interested in IMUs often could not leverage new technical niches due to the barriers in understanding technical requirements and their compatibility with clinical studies. For example, pipelines requiring magnetometers often perform poorly at home due to arbitrary magnetic fields [20], while specialized calibration maneuvers are often neglected or incorrectly followed by patients [21]. To realize the clinical utility of IMUs, simplicity and minimum experimental setup are just as important as accuracy. We consider streamlining the IMU pipeline a pressing need for clinical translation.

Our goals for this study were two-fold. (1) Establish a clinically practical, minimum-setup pipeline for leg-worn IMUs to estimate the knee flexion angle. (2) Determine the concurrent validity of our simple IMU pipeline against optical motion capture (marker-based and markerless) for estimating knee flexion during a large variety of daily living and exercise movements. We hypothesized that knee flexion angle estimated from IMUs would be within the margin of difference between the marker-based and markerless optical systems, and kinematic waveforms would agree strongly across all three systems.

## 2. Materials and Methods

### 2.1. Study Participant Characteristics

We recruited 10 healthy young adults (5 male, 5 female, 21.9 ± 1.9 years old, and body mass index = 23.8 ± 2.4 kg/m^2^) from our local community (e.g., students at our university). A well-cited gait analysis study [1] reported that knee range of flexion in osteoarthritis is reduced by 6° with standard deviations 5–7°. Our power analysis suggested that if we were to perform inferential testing for repeated measures (IMU vs. optical), with α = 0.05 and β = 0.8, a sample size of 10 would be needed to confirm concurrent validity within such a margin of difference. Enrolling 10 healthy volunteers also ensured the feasibility to capture 36 knee movements in a single lab session [10] with 3 concurrent motion tracking systems (IMU + two optical), while maintaining a comparable sample size to similar recent studies [16,17]. We confirmed that each study participant had no current or self-reported lower limb or spine injury in the last 6 months, or a history of any major musculoskeletal or neurological conditions that could interfere with movements. The study was conducted in accordance with the Declaration of Helsinki and approved by the Institutional Review Board of the University of Pennsylvania (Protocol #850424, approved on 27 December 2021). Written informed consent was obtained from all participants involved in the study.

### 2.2. Experimental Procedure and Optical Motion Capture

**Figure 1:**
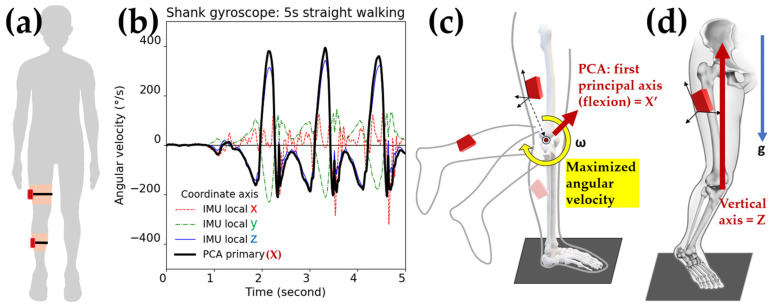
Inertial measurement unit (IMU) physical placement and sensor-to-segment virtual realignment. (**a**) IMU locations on the right leg, one on lateral mid-thigh and the other on lateral mid-shank. (**b**) Example three-dimensional gyroscope data (angular velocity) showing the first 5 s of a trial when participant started from standing position to walk straight. (**c**) We used this 5 s data as input to a principal component analysis (PCA), from which the first principal axis of rotation with maximized angular velocity represented the sagittal knee flexion axis (X’). (**d**) We additionally used gravity during the static pose to identify the vertical axis (Z). Diagrams (**c**,**d**) together depict our workflow for the sensor-to-segment alignment (derived from original work by Cereatti et al. [11], as permitted by a CC BY 4.0 license). In the example shown in (**b**), although the local z axis of this shank-worn IMU (blue solid line) roughly aligned with knee flexion axis, PCA-based transformation also integrated rotations left outside of local z axis (x: red dotted, y: green dashed) to ensure the full knee flexion was captured by the virtually defined primary rotational axis X (thick black solid line).

Study participants wore exercise clothing and standardized running shoes. We attached a pair of research-grade IMUs with accelerometer and gyroscope (Opal V2R, APDM Inc., Portland, OR, USA) to the right leg of each participant, one on lateral mid-thigh and the other on lateral mid-shank (Figure 1a). We wrapped protective elastic bands around participant’s skin before using elastic straps to fasten each IMU securely over the protective bands. Both IMUs recorded at 100 Hz, using ±16 g accelerometer and ±2000 °/s gyroscope amplitude ranges. Signals from the two IMUs were synchronized frame-by-frame with each other (≤0.001 s difference) via Bluetooth wireless streaming to a data receiver (APDM Access Point).

We first recorded a static pose trial with each participant standing in anatomical position, with legs close together and knees fully extended, for IMU and marker-based calibrations. Each participant then performed 36 daily living or exercise movements (Appendix A) [10], where we recorded IMU data for at least 3 successful repetitions of each movement. Participants performed each movement at self-selected speed following instructions, demonstrations, and practice on exercise movements; descriptions on the details of these movements were documented in our previous work [10]. We checked frequently to ensure the IMUs did not slip or loosen on the leg during experiment, and in the few cases that occurred, we adjusted them to the original secured position promptly. We successfully captured IMU data from all 10 participants for most movements (32 of 36); the other 4 movements had data from 8 or 9 participants due to technical issues like dropped Bluetooth streaming signals. We focused our main analysis on 10 representative movements (bolded texts, Table A1), which are either the most essential daily living tasks or the most common knee rehabilitation exercises. We replicated our analysis on the other 26 movements and reported them as secondary data in Supplementary Material S1.

Concurrently with IMUs, we also recorded marker-based motion capture (1.3-megapixel Raptor-E ×10, 12-megapixel Raptor-12 ×2, Motion Analysis Corp., Rohnert Park, CA, USA) and markerless optical motion capture data (2.1-megapixel Optitrack Prime Color, NaturalPoint Inc., Corvallis, OR, USA), both at 100 Hz, following our previously described methods [22]. The two optical motion capture systems were synchronized frame-by-frame to the IMU data receiver via trigger signals through a wired synchronization module (APDM Sync Box v2). For marker-based, we secured 31 skin markers on the pelvis, thighs, shanks, and feet (shoes) and used a constrained kinematic model with 1 degree-of-freedom knee (flexion only) [23]. We chose the constrained knee model so we could use a simple marker set to track valid thigh and shank positions over the large amount of movement trials [23]. For markerless, we recorded synchronized videos and used a deep learning-based human motion tracker (Theia3D v2022.1.0.2309, Theia Markerless Inc., Kingston, ON, Canada) with 3 rotational degrees of freedom at the knee [6]. We used inverse kinematics to calculate knee flexion during each movement for both optical motion capture systems [24].

### 2.3. Knee Flexion Angle Estimation Using Shank and Thigh-Worn IMUs

**Figure 2:**
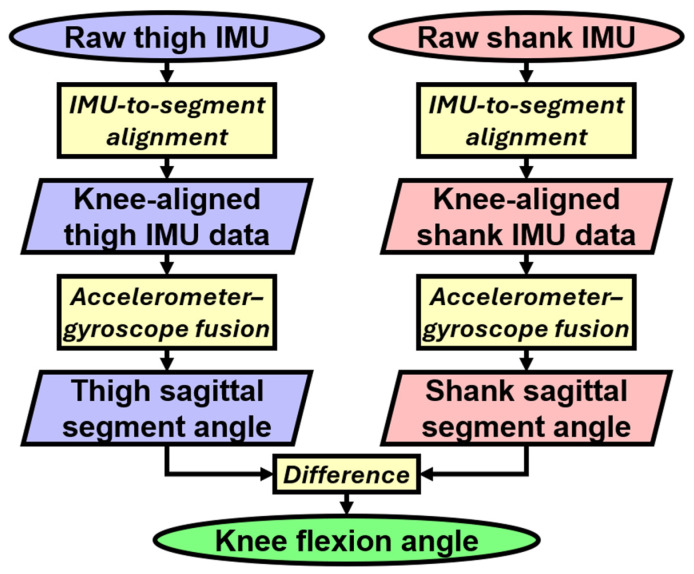
A flowchart that illustrates the overall structure of our IMU data processing pipeline. We applied sensor-to-segment alignment followed by accelerometer–gyroscope fusion independently to the thigh and shank-worn IMU, before combining their results to resolve the knee flexion angle.

We estimated knee flexion angle from thigh and shank-worn IMUs by a combination of sensor-to-segment virtual alignment (Figure 1b–d) [12,25] and accelerometer–gyroscope data fusion algorithms [14]. We applied these algorithms independently to the thigh and shank-worn IMU before combining their results to resolve the knee flexion angle (Figure 2). First, we transformed accelerometer and gyroscope data from each IMU’s local frame (i.e., coordinate system) to an anatomically meaningful knee frame, following a previously established functional alignment technique [25]. Sensor-to-segment coordinate transformation [12] was required because the IMU local frames were orthogonal to the device hardware surface and agnostic to knee anatomy, meaning none of the gyroscope signals readily represent knee flexion. First, to identify the direction of the knee flexion axis (X’) in the IMU local frame, we performed a principal component analysis (PCA) on full three-dimensional gyroscope data to find the axis of rotation with maximized angular velocity (i.e., the first principal component). We performed PCA on the first 5 s of the walking trial, when each participant started from a standing position to walk along a straight path (Figure 1b). We chose this 5 s cutoff for consistency across participants, who stopped straight walking (e.g., turned) at various times after 5 s. Knee motion in these initial 5 s (including gait initiation) remained primarily sagittal; therefore, the first principal axis X’ where shank and thigh rotational velocities were maximized was the axis around which knee flexion occurred (Figure 1c). We ensured +X’ was lateral (i.e., not medial) by enforcing its direction in IMU’s local z axis to be positive, negating it if negative. For our IMUs, local z (Figure 1b) pointed away from the surface that contacted the leg, which was lateral because we placed the IMUs *generally* on the lateral side of both thigh and shank (Figure 1a). Second, we identified the knee proximo-distal axis (Z) using the standing pose, when the thigh and shank’s long axes are both assumed to be vertically aligned with the static accelerometer signal (i.e., gravity) (Figure 1d). Third, we calculated the antero-posterior axis (Y) as the cross product of Z and X’ axes, then recalculated the final orthogonal X axis to be the cross product of Y and Z axes. We established IMU-to-segment frame transformations independently for the shank and thigh, then transformed accelerometer and gyroscope data to their segment virtual frames, where their X component was the projected sagittal knee flexion axis, Y was antero-posterior, and Z was proximo-distal along the segment long axis [25]. We performed sensor-to-segment alignment once per participant (pose and walking trial) and applied the transformation to all movement trials from the same participant, with the assumption that each IMU did not move relative to its attached segment within each experimental session.

We estimated the orientations of each IMU by feeding the sensor-to-segment transformed data into a commonly used, open-source accelerometer–gyroscope fusion algorithm (“Madgwick filter”; x-io Technologies Limited, Bristol, UK) [14]. We chose this open-source algorithm (specifically, its no-magnetometer version) over vendor-supplied proprietary fusion filter because it makes our paradigm applicable to other IMUs, especially low-cost devices that do not have a magnetometer. The working principle of accelerometer–gyroscope fusion is well-documented [13,14]. Briefly, we integrated angular velocity from gyroscope to estimate orientation (angle) and used linear acceleration from accelerometer to correct for sensor tilt relative to gravity and the drift accumulated from gyroscope noise. We set the gain of the Madgwick filter at β = 0.5, which was the default; our preliminary sensitivity analysis revealed that our fusion outcome was not sensitive to the gain value of choice (Supplementary Material S2: Figure S1). We noticed during pilot testing that our filter took up to 5 s to converge from an arbitrary initial orientation guess. This led to irregular IMU tracking artifacts during the initial seconds of data, especially in brief trials with fast movements (Supplementary Material S2: Figure S2). To ensure correct estimation for the whole trials, we prepended 5 s of virtual IMU data that “froze” at the (sensor-to-segment transformed) linear acceleration of the start of each trial, with zero angular velocity. This “freeze” allowed our algorithm to coverage to its true starting orientation in the knee segment frame in time. Output of the fusion filter was the orientation of each IMU relative to the global inertial frame, expressed as quaternions.

We converted the fusion-estimated IMU orientation quaternions into Euler angles, where rotation around the segment-aligned first principal axis (X) represented the sagittal segment angle. We calculated the knee flexion angle as the difference between the sagittal shank angle and sagittal thigh angle, with matched lateral +X signs [25] (Figure 2). To maximize the reproducibility of our IMU pipeline by other researchers, we have made our IMU data processing code openly available in a public repository, free to access via DOI link at https://doi.org/10.5281/zenodo.20548127.

### 2.4. Data Analysis and Statistics

We computed the knee flexion angle during all movement repetitions from all 3 motion tracking systems. For cyclic gaits (walking and running), we identified gait cycles using ground reaction force and heel position. For other motion trials, we manually defined start and stop events that represent the weight-bearing phase, as we previously detailed [10]. We computed Pearson correlation coefficient (R_xy_) twice, one for IMU-based versus marker-based knee flexion estimate, and again separately for IMU versus the markerless optical estimate. R_xy_ quantifies the shape agreement of two knee flexion waveforms on each repetition. We also computed root-mean-square difference (RMSD) for IMU versus marker-based and separately for IMU versus markerless optical, which quantifies their mean magnitude differences. We previously reported the concurrent validity between our marker-based and markerless optical systems from the same experiment [22]; therefore, we did not include that comparison in our current work.

We calculated R_xy_ and RMSD between the IMU and optical systems on each individual movement repetition, before averaging across repetitions within each participant. We then calculated the mean across 10 participants to determine the group-wise waveform agreement (R_xy_) and magnitude difference (RMSD) for IMU versus marker-based and IMU versus markerless optical estimates. According to Schober et al. [26], we defined that R_xy_ ≥ 0.7 suggests a strong correlation between two systems, and R_xy_ ≥ 0.9 suggests a very strong correlation. According to recommendations in the literature [27,28] and our previous work [22], we defined that the joint angle magnitude difference is minimal if RMSD ≤ 5°.

## 3. Results

### 3.1. IMU Tracking Versus Marker-Based Motion Capture

**Figure 3:**
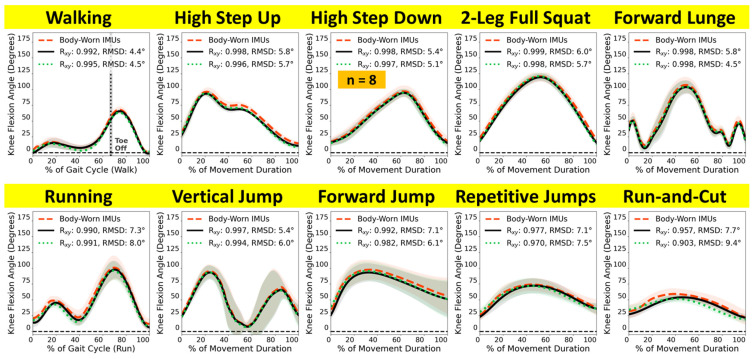
Knee flexion angle estimated from leg-worn IMUs (red dashed) compared to marker-based (black solid) and markerless optical motion capture (green dotted) during 10 representative movements. Waveforms = group mean (line) ± 1 standard deviation (shadow). (**Top**) IMUs had very strong agreements to both optical systems during gait, stair navigation, and high knee flexion movements. (**Bottom**) Differences between IMUs and the optical systems were larger for faster athletic movements like running and jumping. Walking and running were analyzed over a gait cycle (between heel strikes). Start and end definition for the other movements were detailed in our prior work [10]. IMU data for high step down were lost for 2 participants due to technical issues (e.g., dropped Bluetooth streaming signals). For full numerical data supporting these results, see Table 1.

Knee flexion angle estimated from thigh and shank-worn IMUs showed generally good concurrent validity to marker-based estimates, especially during slower movements (Figure 3, IMU: red dashed, marker-based: black solid; Table 1). Correlation of waveforms over a walking cycle was R_xy_ = 0.992, and their magnitude difference met our minimal criterion (RMSD = 4.4°). Likewise, during other slower movements like stair navigation (step up, step down) and high knee flexion tasks (squat, lunge), waveform correlations between IMU and marker-based were very strong (R_xy_ ≥ 0.998), with magnitude differences just above our minimal criterion (RMSD: 5.4–6.0°). Differences were relatively larger for faster movements. Specifically, waveform correlation over a running gait cycle was R_xy_ = 0.990, and their magnitude difference exceeded our criterion of minimal (RMSD = 7.3°). Waveform correlations were very strong for vertical jump (R_xy_ = 0.997) and forward jump (R_xy_ = 0.992), while relatively less strong for repetitive jumps (R_xy_ = 0.977) and the run-and-cut maneuver (R_xy_ = 0.957). Magnitude difference for vertical jump (RMSD = 5.4°) was comparable to slower movements, but larger for forward jump, repetitive jumps, and run-and-cut (RMSD = 7.1–7.7°). The most dynamic movement, run-and-cut, observed the largest magnitude difference (RMSD = 7.7°) and relatively the lowest waveform agreement (R_xy_ = 0.957), yet still met our a priori criterion of very strong correlation.

### 3.2. IMU Tracking Versus Markerless Optical Motion Capture

The concurrent validity between IMU and our markerless optical system was generally the same as IMU versus marker-based as follows: very strong waveform agreement and small magnitude differences, especially for slower movements (Figure 3, IMU: red dashed, markerless optical: green dotted; Table 1). For a walking cycle, waveform correlation was R_xy_ = 0.995, and magnitude difference was RMSD = 4.5°. For other slower movements (stair navigation and high knee flexion), waveform agreements were again very strong (R_xy_ ≥ 0.996), and magnitude differences were just above the minimal threshold (RMSD = 5.1–5.7°) except for forward lunge, which met the minimal criterion (RMSD = 4.5°). For a running gait cycle, waveform agreement was almost the same as versus marker-based (R_xy_ = 0.991) while magnitude difference was marginally larger (RMSD = 8.0°). Waveform agreements were very strong for vertical jump (R_xy_ = 0.994) but slightly lower than the agreements with marker-based for other fast movements (R_xy_ = 0.903–0.982); magnitude differences varied in the above-minimal range (RMSD = 6.0–9.4°). Run-and-cut again observed the largest RMSD (9.4°) and the lowest waveform agreement (R_xy_ = 0.903), both relatively worse than the comparability between IMU and marker-based. Overall, across the 10 movements, walking consistently showed the best match between IMU and the two optical systems, while run-and-cut matched consistently the worst, followed by running.

## 4. Discussion

Our goals for this study were to (1) establish a clinically practical, minimum-setup pipeline for leg-worn IMUs to estimate knee flexion and (2) determine its concurrent validity to optical motion capture during various knee movements. Results supported our hypotheses that knee flexion angle estimated using IMUs is within the margin of difference between marker-based and markerless optical motion capture and that kinematic waveforms agreed strongly across all three systems. Magnitude differences met our preset criterion of minimal during walking (<5°) and only slightly exceeded this criterion during other slow movements (5–6°) but were larger for faster sports-related movements (5.4–9.4°). Good agreements during slower movements like gait confirm similar IMU studies and support IMU as a potentially suitable tool for tracking daily living knee mobility in chronic disease populations like osteoarthritis. Conversely, it is inconclusive whether IMUs can accurately track real-world athletic knee motion relevant to injuries. Our three-way concurrent comparison between conventional marker-based motion capture, low-burden markerless motion capture, and wearable IMU motion tracking across slow and fast movements provide a first-of-kind benchmark for researchers, clinicians, and athletic trainers to evaluate the pros and cons of each platform and choose the best system for knee motion analysis based on their unique needs.

Our first finding was that IMUs track knee flexion with comparable results to motion capture during daily living tasks like walking, stair navigation, and high knee flexion movements (squat and lunge) (Figure 3, top row). IMU-estimated knee flexion showed very strong waveform agreement (R_xy_ > 0.99) to both marker-based and markerless motion capture. Notably, the magnitude difference during walking (RMSD ≤ 4.5°) met our preset criterion of minimal difference (5°). We confirmed that these favorable results on walking were not biased by our decision to use the same walking trial for sensor alignment, as alternative alignment trials produced largely equivalent results (Supplementary Material S2: Figure S3). In our previous analysis on this same experiment [22], comparing between markerless and marker-based systems, we saw similar waveform agreement (R_xy_ = 0.965 ± 0.019) and magnitude difference (RMSD = 3.9° ± 2.1°) during walking. IMU-estimated knee flexion is therefore within the margin of uncertainty between our two optical motion capture systems. Considering together with our previous work, markerless videos and leg-worn IMUs are both suitable for quantifying knee flexion and offer practical benefits over conventional marker-based systems. Markerless optical motion capture is a good choice for in-clinic motion analysis where laboratory space is available, due to its low burden on patients and excellent inter-session reliability [7,29]. IMUs are a good choice for clinical studies where laboratory space is limited, or when real-world continuous monitoring is essential. Our current data indicate that IMU-based high fidelity knee flexion tracking is available to most activities that patients with limited knee mobility would perform. Therefore, clinical researchers can leverage IMUs to track the frequency of functional knee movements in daily life for a better understanding of the total mechanical exposure imposed on diseased knees.

IMU-based knee flexion measured during faster movements strongly correlated with the optical systems (R_xy_ > 0.9), but RMSDs exceeded our 5° minimal difference criterion (Figure 3, bottom row). In our previous comparison between markerless and marker-based [22], waveform agreement was strong and magnitude difference was minimal for running (R_xy_ = 0.944, RMSD = 4.8°) and vertical jump (R_xy_ = 0.997, RMSD = 3.4°), albeit less so for run-and-cut (R_xy_ = 0.877, RMSD = 5.9°). The RMSD for IMU-estimated knee flexion slightly exceeded these difference margins (Figure 3). However, because soft-tissue artifacts [27] can induce up to 8.3° error in marker-estimated knee flexion compared to ground-truth biplane fluoroscopy [28], RMSD < 8° should be considered within the range of uncertainty when using marker-based motion capture as reference. Our IMU-based estimates were within this ~8° uncertainty range versus marker-based across all 10 representative movements (RMSD ≤ 7.7°; Figure 3). The differences between IMU and motion capture were also consistent across participants and movement repetitions. Our ancillary data suggest that the *inter-subject* standard deviation of RMSD was generally in the 2–3° range (Table 1), while the *intra-subject* variability of RMSD (e.g., step-to-step) was <1° for slower movements and 1–2.5° for the faster ones (Supplementary Material S2: Table S1). We are not aware of any concurrent validation study between leg-worn IMUs and biplane fluoroscopy, and it is beyond our scope to speculate whether IMU or marker-based knee flexion angle is closer to ground truth during fast movements. The decision to trust IMUs for tracking knee flexion during those movements should thus be left to the confidence investigators have in optical motion capture fidelity.

Our results compare favorably to other IMU-based studies. Prior IMU studies that quantified knee flexion during walking and running reported RMSD < 4° [13,16,17,30]. Our results matched those findings on walking (RMSD = 4.4°), although we saw a larger difference on running (7.3°). A recent systematic review reported minimal clinically important changes in knee flexion to be 6.4° for pain and 5.6° for function in non-surgical knee osteoarthritis patients [31]. Our inter-system RMSD of 4.4° for walking falls under these thresholds, suggesting IMUs can detect clinically meaningful knee flexion changes akin to gait analysis. Studies on other knee exercises reported highly variable inter-system differences. For example, RMSDs between IMU and marker-based during run-and-cut ranged from 1.1° [32] to 12.1° [9]; our finding (7.7°) falls within this range. A novel finding from our study was that the IMU-to-markerless differences were almost the same as IMU-to-marker-based (Figure 3). While each system estimates knee flexion comparably, especially for slower movements, their unique practical strengths and limitations [9] should be evaluated when selecting the most suitable system for research or clinical purposes.

By demonstrating sufficient concurrent validity to estimate knee flexion, we established a simple, streamlined IMU pipeline with as few experimental requirements as we had to keep. Our algorithm requires no magnetometer; thus, it is compatible with lower-cost IMUs and supports deploying sensors to more patients in larger clinical cohorts. This also extends the monitoring duration by reducing data size and battery use, so clinicians can record robust day-long data without burdening patients with sensor charging or reattachment. Our sensor-to-segment alignment uses readily available walking data and needs no specialized calibration, eliminating the need for a patient to memorize and perform them. Experimental setups like device type, cost, storage, charging, attachment, and calibration are easily fulfilled in idealized IMU development tests but can be easily violated in clinical deployment, in our experience. Our “minimalist” approach makes our pipeline accessible to non-IMU experts who wish to leverage them, especially clinical researchers. Instead of advancing to a new IMU technical niche, our work innovates and contributes practically by supporting the clinical translation of state-of-the-art IMU methods, making them compatible with and ready to be integrated into scalable clinical studies.

Our findings should be interpreted with several limitations. We selected a relatively small sample size of 10 healthy volunteers to ensure the feasibility of capturing 36 movements in one experiment [10] with three concurrent motion trackers. However, our sample size was supported by a power analysis and comparable to similar recent studies [16,17]. We prioritized knee flexion because non-sagittal knee kinematics estimated by marker-based motion capture are susceptible to skin artifacts [28] and not reliable references for cross-validation. We defined full knee extension—required for defining the virtual segment frames (Figure 1d)—using a standing pose trial. Standing with a straight knee may be difficult for patients with osteoarthritis due to painful limited motion. Updated experimental procedures to establish segment coordinate frames are needed, and updated IMU algorithms should be revalidated for patients with symptomatic knee osteoarthritis. While we expect our findings to generalize to clinical populations and natural environments, namely IMUs would track a patient’s knee as well as a healthy knee outside the lab, confirmation directly on the cohort of interest and in daily living settings will be valuable.

## 5. Conclusions

We developed a simple, minimum-setup pipeline that tracks knee flexion angles with thigh and shank-worn IMUs, demonstrating similar accuracy to concurrent marker-based and markerless optical motion capture. Good agreements between IMUs and optical systems during essential daily living tasks like gait, stair navigation, and high knee flexion movements confirm similar studies and suggest IMUs are potentially suitable for monitoring the real-world mobility of knees with chronic diseases. Conversely, the accuracy of IMUs to track real-world athletic knee motion is inconclusive and warrants further research. Our validated simple IMU pipeline streamlines the experimental setup and makes it more practical and compatible with clinical research. Future work should seek best practices for IMU wearing and improve the feasibility, reliability, and validity of body-worn IMU continuous tracking on real-world knee motion. IMUs present an exciting platform for clinicians to monitor real-world knee function in both physically active and impaired populations, for deeper insights into the cumulative impacts of knee motion on joint overuse injuries and chronic degenerative diseases.

## Figures and Tables

**Table 1:** Descriptive statistics on group-wise trends of waveform agreement (R_xy_) and magnitude difference (RMSD) between knee flexion estimated from IMUs vs. optical motion capture, based on the per-subject average of these metrics. SD, standard deviation; IQR, inter-quartile range.

**IMU vs. Marker-Based: R_xy_ and RMSD in Knee Flexion (Based on Per-Subject Average), *n* = 10**						
**Movement**	**Pearson Correlation Coefficient, R_xy_**			**Root-Mean-Square Difference, RMSD**		
	**Mean ^1^**	**SD**	**IQR**	**Mean ^1^**	**SD**	**IQR**
Walking	0.992	0.004	0.003	4.4°	2.0°	1.9°
High Step Up	0.998	0.001	0.002	5.8°	3.7°	4.7°
High Step Down *	0.998	0.001	0.002	5.4°	3.2°	3.6°
2-Leg Full Squat	0.999	0.001	0.001	6.0°	2.7°	3.7°
Forward Lunge	0.998	0.001	0.001	5.8°	2.9°	3.8°
Running	0.990	0.007	0.009	7.3°	3.7°	5.1°
Vertical Jump	0.997	0.002	0.002	5.4°	2.2°	1.2°
Forward Jump	0.992	0.006	0.007	7.1°	3.1°	5.0°
Repetitive Jumps	0.977	0.030	0.011	7.1°	3.6°	4.8°
Run-and-Cut	0.957	0.018	0.029	7.7°	5.7°	4.2°
**IMU vs. Markerless Optical: R_xy_ and RMSD in Knee Flexion (Based on Per-Subject Average), *n* = 10**						
**Movement**	**Pearson Correlation Coefficient, R_xy_**			**Root-Mean-Square Difference, RMSD**		
	**Mean ^2^**	**SD**	**IQR**	**Mean ^2^**	**SD**	**IQR**
Walking	0.995	0.003	0.004	4.5°	2.9°	5.4°
High Step Up	0.996	0.003	0.002	5.7°	3.0°	4.1°
High Step Down *	0.997	0.002	0.002	5.1°	3.3°	6.3°
2-Leg Full Squat	0.998	0.002	0.002	5.7°	2.2°	2.2°
Forward Lunge	0.998	0.001	0.000	4.5°	2.8°	4.9°
Running	0.991	0.007	0.006	8.0°	3.7°	6.8°
Vertical Jump	0.994	0.002	0.002	6.0°	1.9°	3.3°
Forward Jump	0.982	0.015	0.019	6.1°	2.7°	2.4°
Repetitive Jumps	0.970	0.014	0.013	7.5°	3.4°	3.3°
Run-and-Cut	0.903	0.079	0.081	9.4°	4.9°	4.7°

^1^ Correspond to data reported in Figure 3 and Section 3.1 of the Results text. ^2^ Correspond to data reported in Figure 3 and Section 3.2 of the Results text. * *n* = 8 due to lost trials caused by technical issues (e.g., dropped Bluetooth streaming signals).

## Data Availability

The original IMU data and processing code presented in the study are openly available in the Zenodo public repository at https://doi.org/10.5281/zenodo.20548127.

## Supplementary Materials

The following supporting information can be downloaded at https://www.mdpi.com/article/10.3390/s26123704/s1, Supplementary Material S1: Knee flexion angle estimated from leg-worn IMUs compared to marker-based and markerless optical systems across a total of 36 movements; Supplementary Material S2: Ancillary Data and Sensitivity Analyses.

## Abbreviations

The following abbreviations are used in this manuscript:

IMU	Inertial measurement unit
RMSD	Root-mean-square difference
PCA	Principal component analysis

## Appendix A. 36 Movement Trials Listed in the Order of Experimental Data Collection

Table A1 below describes the names of 36 movement trials each participant performed and the four “modules” they belong in, listed in the order of experimental data collection. Bolded texts in Table A1 annotate the 10 movements reported as main findings in Results. Results for the other 26 movements are reported in Supplementary Material S1. Detailed trial definitions can be found in our prior work [10].

**Table A1:** The 36 movement trials, listed in the order of experimental data collection.

Module 1	Module 2	Module 3	Module 4
Exercise 1–11	Exercise 12–21	Exercise 22–29	Exercise 30–36
Heel raises	**Running**	1-leg squat (60-degree)	1-leg drop landing
**Walking**	2-leg squat (60-degree)	1-leg squat (full depth)	1-leg drop vertical hop
Low step up (10 cm)	**2-leg squat (full depth)** ***“2-Leg Full Squat”***	Bulgarian squat	1-leg repeat forward hops
Low step down (10 cm)	1-leg decline squat	1-leg countermovement hop	1-leg fast forward hops
**High step up (20 cm)**	Sumo squat	**2-leg repeat forward jumps** ***“Repetitive Jumps”***	1-leg repeat lateral hops
**High step down (20 cm)**	1 s Spanish squat	2-leg fast forward jumps	1-leg fast lateral hops
**Lunge** ***“Forward Lunge”***	**Run-and-cut**	2-leg repeat lateral jumps	Alternating split jumps
**2-leg countermovement jump** ***“Vertical Jump”***	1-leg maximal forward hop	2-leg fast lateral jumps	
2-leg drop landing	Run-and-stop		
2-leg drop vertical jump	Sports movement jump		
**2-leg maximal forward jump** ***“Forward Jump”***			

**Bolded texts** indicate the 10 representative movements in our main analysis. Analysis on the other 26 movements are reported as secondary data in Supplementary Material S1. Non-italicized texts indicate the original trial names defined in our prior work [10]. *Italicized texts* indicate the shortened “alternative trial names” used in Figure 3.
