# Supplementary material for: A Simple Minimum-Setup Pipeline for Using Leg-Worn Inertial Sensors to Track Knee Flexion: Validation on 10 Movements
Source: Sensors (Basel). 2026 Jun 10;26(12):3704. doi: 10.3390/s26123704 (PMC13307479; doi:10.3390/s26123704)
Supplement: Supplementary file 1 [file sensors-26-03704-s001.zip › sensors-4323304 _Supplementary Material 1.pdf]

Supplementary Material 1: Knee flexion angle estimated from leg-worn IMUs compared to marker-based and markerless optical systems across a total of 36 movements

The secondary data presented here are extended from the same biomechanical analysis we performed on the 10 movements presented in the main article. Each participant performed all 36 movements during the experiment of a larger study, in the order identical to the Supplementary Figures presented below (i.e., heel raises, walking, etc.) **Titles highlighted in teal color** annotate the 10 representative movements we included in the main article. Waveforms = group mean (line) = ± 1 standard deviation (shade) for **body-worn IMUs (red dashed)**, **marker-based (black solid)**, and **markerless optical (green dotted)** systems. Start and end definition for the other movements were detailed in our prior work [see citation ^]. IMU data for some participants were lost for low step down (1 lost), high step down (2 lost), 2-leg drop vertical jump (1 lost), and 2-leg fast-speed lateral jumps (1 lost) due to technical issues, e.g., dropped Bluetooth streaming signals. Such cases are labeled with their special sample size ("n = #") in the following Supplementary Figures.

[^] Song, K.; Scattone Silva, R.; Hullfish, T.J.; Silbernagel, K.G.; Baxter, J.R. Patellofemoral joint loading progression across 35 weightbearing rehabilitation exercises and activities of daily living. *Am. J. Sports Med.* **2023,** *51,* 2110–2119. https://doi.org/10.1177/03635465231175160. (Citation [10] in the main article.)

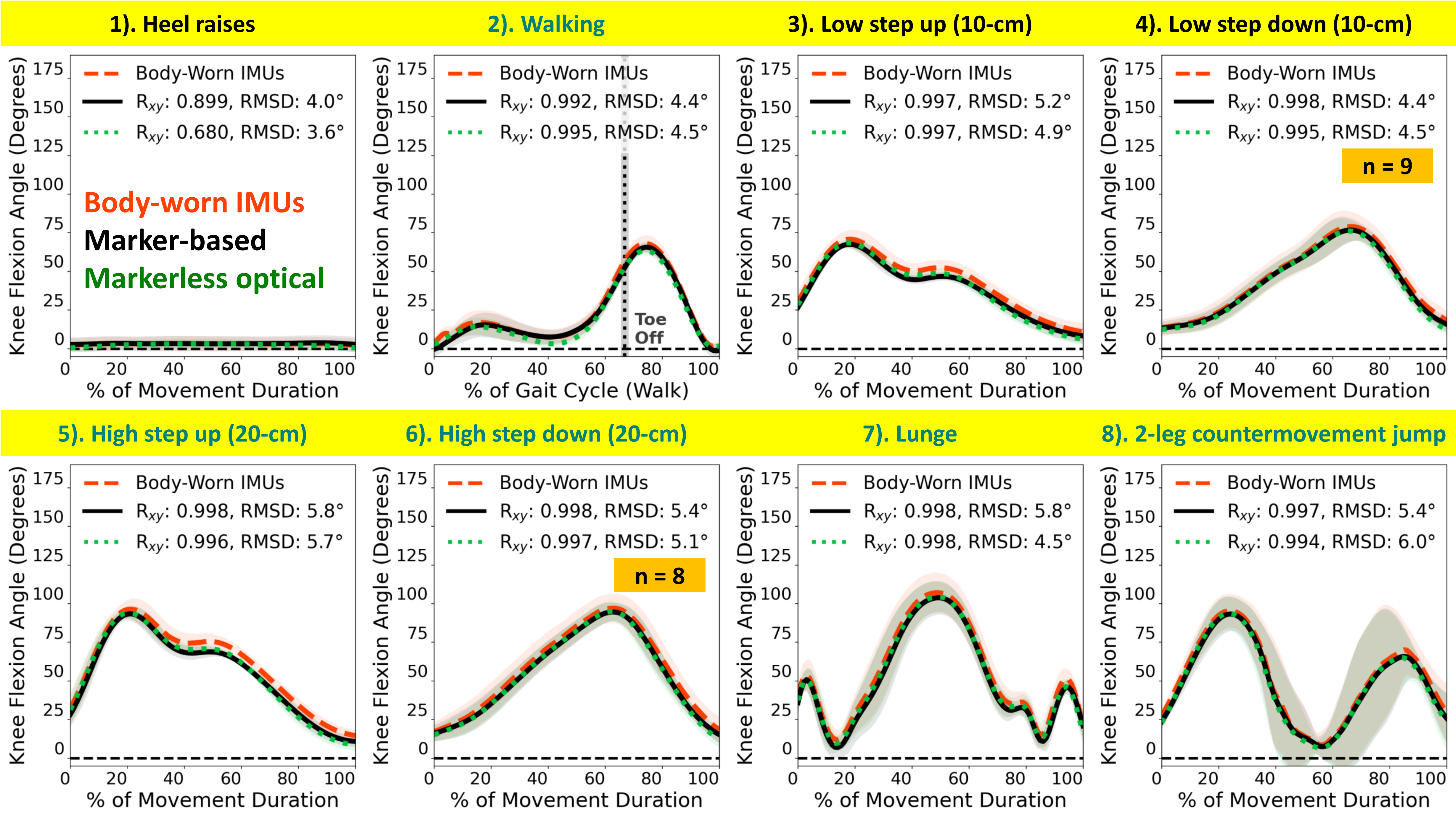
1). Heel raises
2). Walking
3). Low step up (10-cm)
4). Low step down (10-cm)
Body-Worn IMUs
R_{xy}: 0.899, RMSD: 4.0°
R_{xy}: 0.680, RMSD: 3.6°
Body-worn IMUs
Marker-based
Markerless optical
Knee Flexion Angle (Degrees)
% of Movement Duration
R_{xy}: 0.992, RMSD: 4.4°
R_{xy}: 0.995, RMSD: 4.5°
Toe Off
% of Gait Cycle (Walk)
R_{xy}: 0.997, RMSD: 5.2°
R_{xy}: 0.997, RMSD: 4.9°
R_{xy}: 0.998, RMSD: 4.4°
R_{xy}: 0.995, RMSD: 4.5°
n = 9
5). High step up (20-cm)
6). High step down (20-cm)
7). Lunge
8). 2-leg countermovement jump
R_{xy}: 0.998, RMSD: 5.8°
R_{xy}: 0.996, RMSD: 5.7°
R_{xy}: 0.998, RMSD: 5.4°
R_{xy}: 0.997, RMSD: 5.1°
n = 8
R_{xy}: 0.998, RMSD: 5.8°
R_{xy}: 0.998, RMSD: 4.5°
R_{xy}: 0.997, RMSD: 5.4°
R_{xy}: 0.994, RMSD: 6.0°

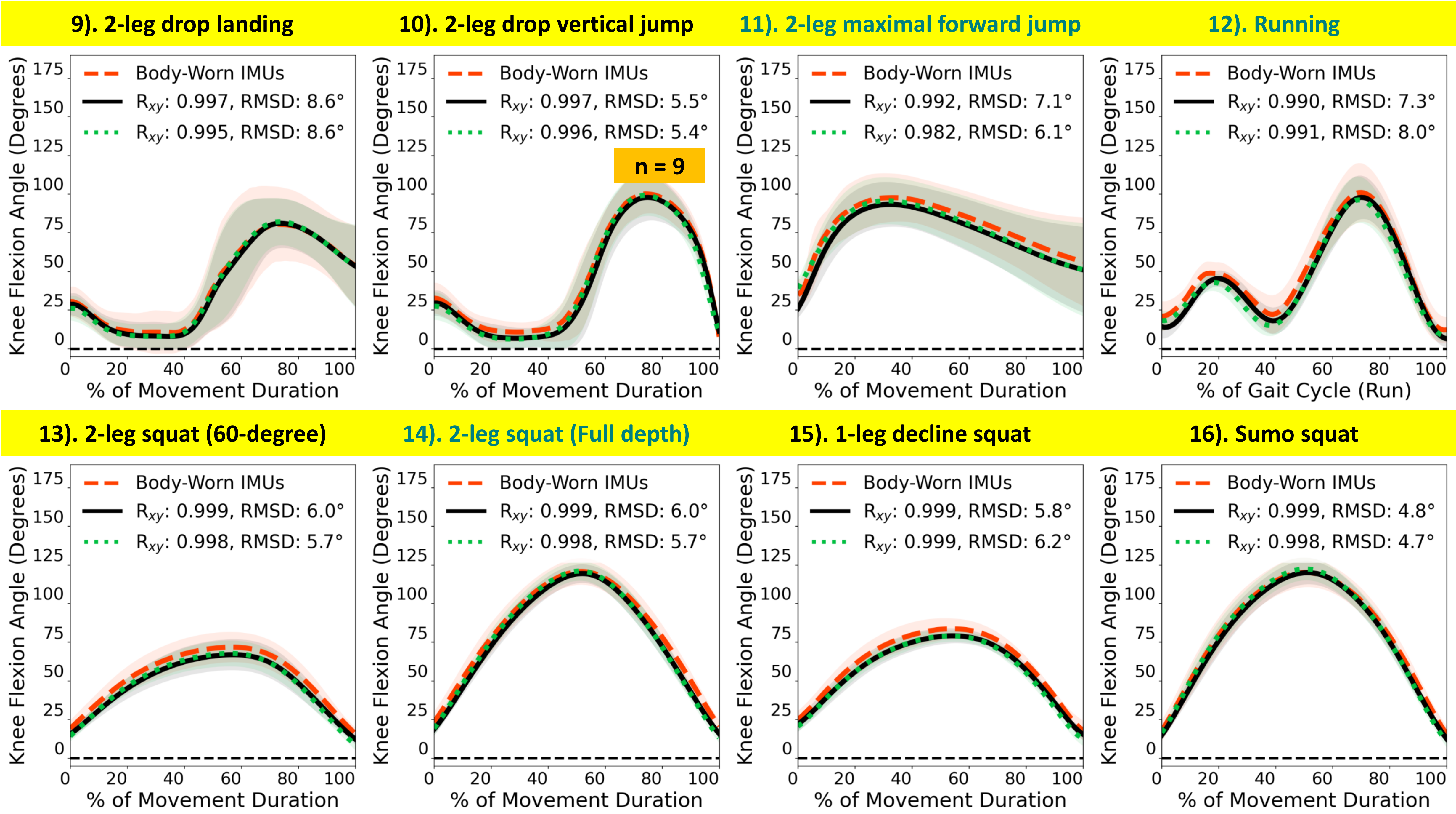

9). 2-leg drop landing
Body-Worn IMUs
Rxy: 0.997, RMSD: 8.6°
Rxy: 0.995, RMSD: 8.6°
Knee Flexion Angle (Degrees)
% of Movement Duration
10). 2-leg drop vertical jump
Body-Worn IMUs
Rxy: 0.997, RMSD: 5.5°
Rxy: 0.996, RMSD: 5.4°
n = 9
Knee Flexion Angle (Degrees)
% of Movement Duration
11). 2-leg maximal forward jump
Body-Worn IMUs
Rxy: 0.992, RMSD: 7.1°
Rxy: 0.982, RMSD: 6.1°
Knee Flexion Angle (Degrees)
% of Movement Duration
12). Running
Body-Worn IMUs
Rxy: 0.990, RMSD: 7.3°
Rxy: 0.991, RMSD: 8.0°
Knee Flexion Angle (Degrees)
% of Gait Cycle (Run)
13). 2-leg squat (60-degree)
Body-Worn IMUs
Rxy: 0.999, RMSD: 6.0°
Rxy: 0.998, RMSD: 5.7°
Knee Flexion Angle (Degrees)
% of Movement Duration
14). 2-leg squat (Full depth)
Body-Worn IMUs
Rxy: 0.999, RMSD: 6.0°
Rxy: 0.998, RMSD: 5.7°
Knee Flexion Angle (Degrees)
% of Movement Duration
15). 1-leg decline squat
Body-Worn IMUs
Rxy: 0.999, RMSD: 5.8°
Rxy: 0.999, RMSD: 6.2°
Knee Flexion Angle (Degrees)
% of Movement Duration
16). Sumo squat
Body-Worn IMUs
Rxy: 0.999, RMSD: 4.8°
Rxy: 0.998, RMSD: 4.7°
Knee Flexion Angle (Degrees)
% of Movement Duration

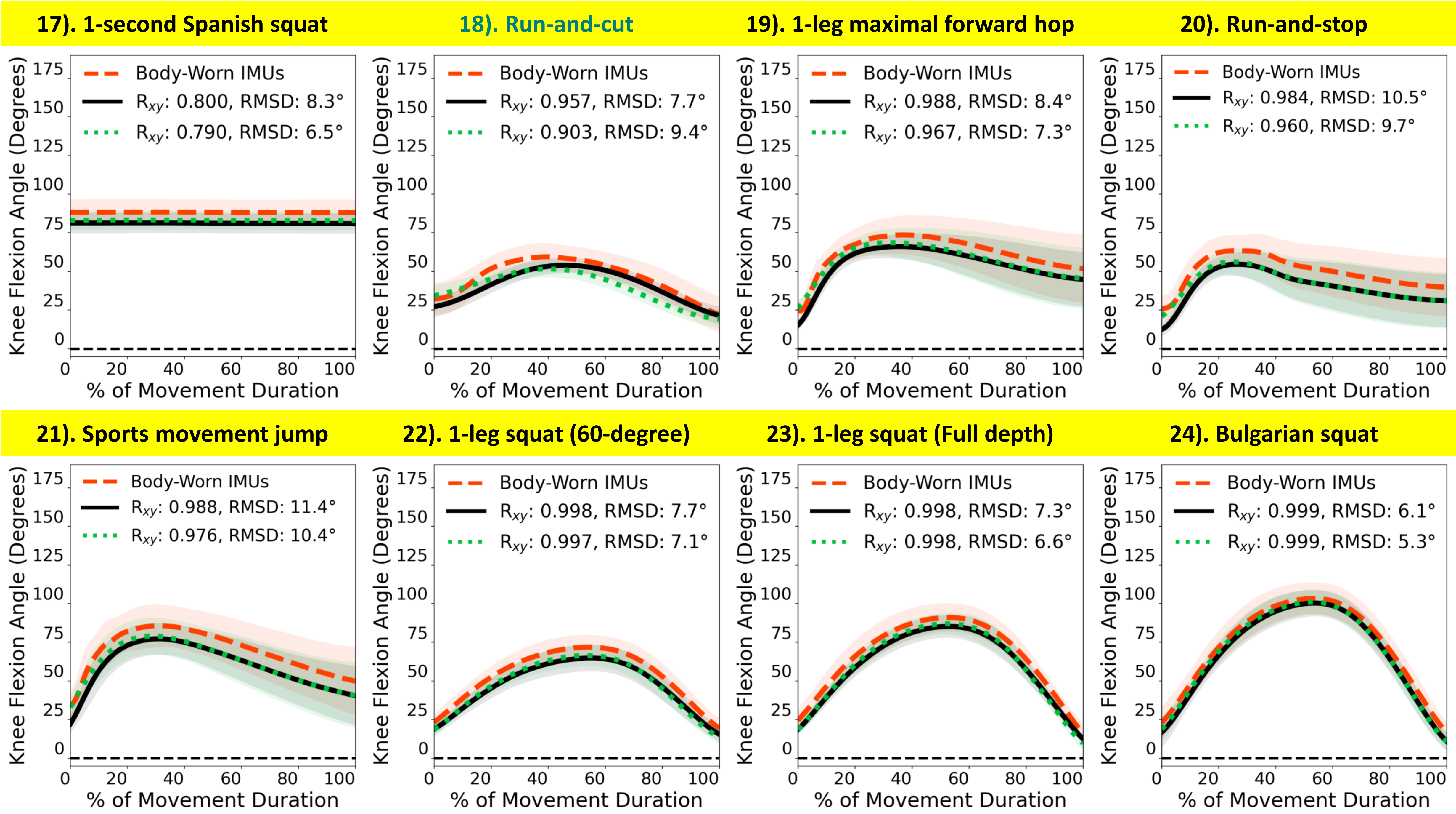

17). 1-second Spanish squat
Body-Worn IMUs
Rxy: 0.800, RMSD: 8.3°
Rxy: 0.790, RMSD: 6.5°
18). Run-and-cut
Body-Worn IMUs
Rxy: 0.957, RMSD: 7.7°
Rxy: 0.903, RMSD: 9.4°
19). 1-leg maximal forward hop
Body-Worn IMUs
Rxy: 0.988, RMSD: 8.4°
Rxy: 0.967, RMSD: 7.3°
20). Run-and-stop
Body-Worn IMUs
Rxy: 0.984, RMSD: 10.5°
Rxy: 0.960, RMSD: 9.7°
21). Sports movement jump
Body-Worn IMUs
Rxy: 0.988, RMSD: 11.4°
Rxy: 0.976, RMSD: 10.4°
22). 1-leg squat (60-degree)
Body-Worn IMUs
Rxy: 0.998, RMSD: 7.7°
Rxy: 0.997, RMSD: 7.1°
23). 1-leg squat (Full depth)
Body-Worn IMUs
Rxy: 0.998, RMSD: 7.3°
Rxy: 0.998, RMSD: 6.6°
24). Bulgarian squat
Body-Worn IMUs
Rxy: 0.999, RMSD: 6.1°
Rxy: 0.999, RMSD: 5.3°
Knee Flexion Angle (Degrees)
% of Movement Duration

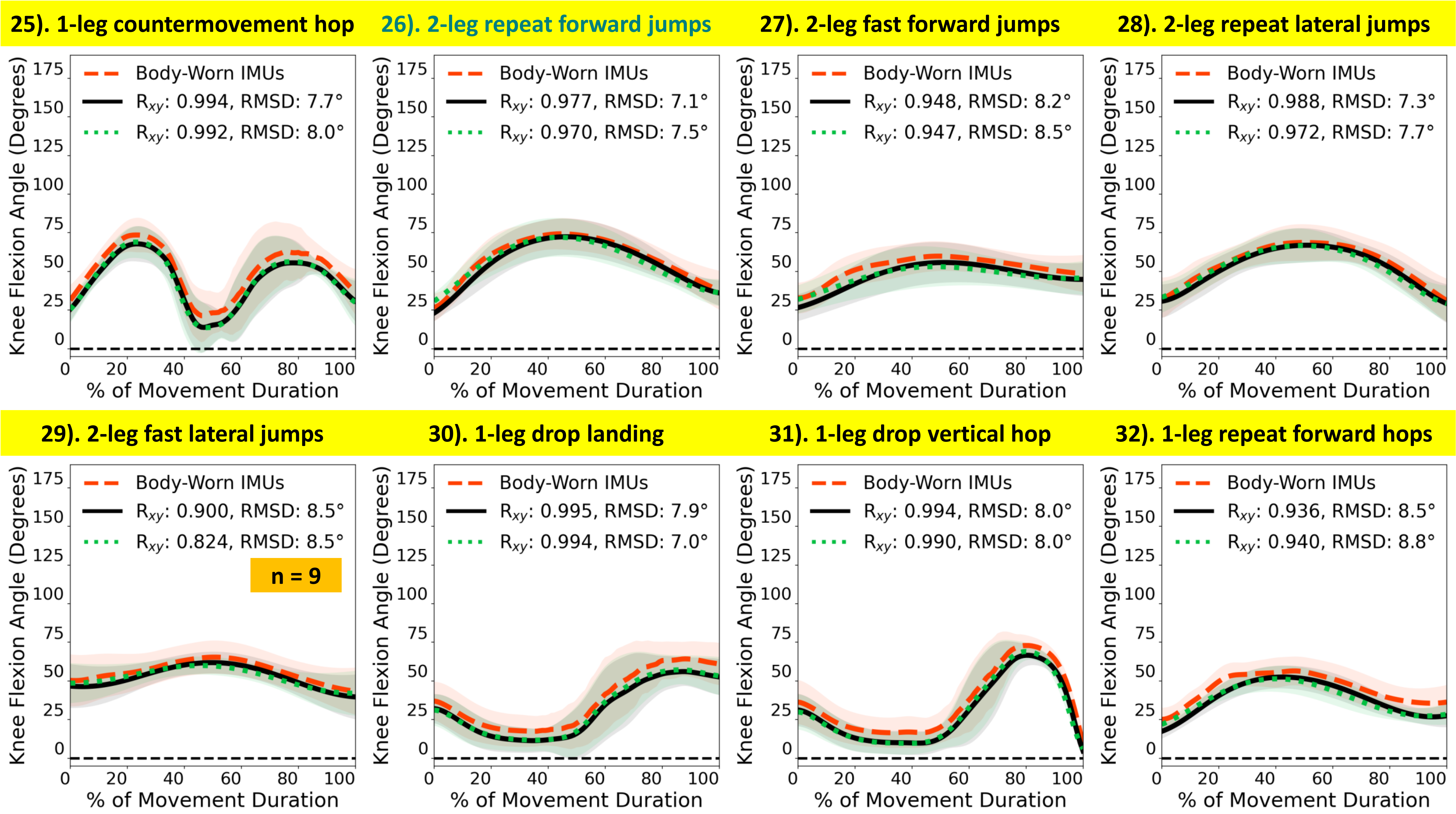
25). 1-leg countermovement hop
Body-Worn IMUs
Rxy: 0.994, RMSD: 7.7°
Rxy: 0.992, RMSD: 8.0°
26). 2-leg repeat forward jumps
Body-Worn IMUs
Rxy: 0.977, RMSD: 7.1°
Rxy: 0.970, RMSD: 7.5°
27). 2-leg fast forward jumps
Body-Worn IMUs
Rxy: 0.948, RMSD: 8.2°
Rxy: 0.947, RMSD: 8.5°
28). 2-leg repeat lateral jumps
Body-Worn IMUs
Rxy: 0.988, RMSD: 7.3°
Rxy: 0.972, RMSD: 7.7°
29). 2-leg fast lateral jumps
Body-Worn IMUs
Rxy: 0.900, RMSD: 8.5°
Rxy: 0.824, RMSD: 8.5°
n = 9
30). 1-leg drop landing
Body-Worn IMUs
Rxy: 0.995, RMSD: 7.9°
Rxy: 0.994, RMSD: 7.0°
31). 1-leg drop vertical hop
Body-Worn IMUs
Rxy: 0.994, RMSD: 8.0°
Rxy: 0.990, RMSD: 8.0°
32). 1-leg repeat forward hops
Body-Worn IMUs
Rxy: 0.936, RMSD: 8.5°
Rxy: 0.940, RMSD: 8.8°
Knee Flexion Angle (Degrees)
% of Movement Duration
0
25
50
75
100
125
150
175
0
20
40
60
80
100

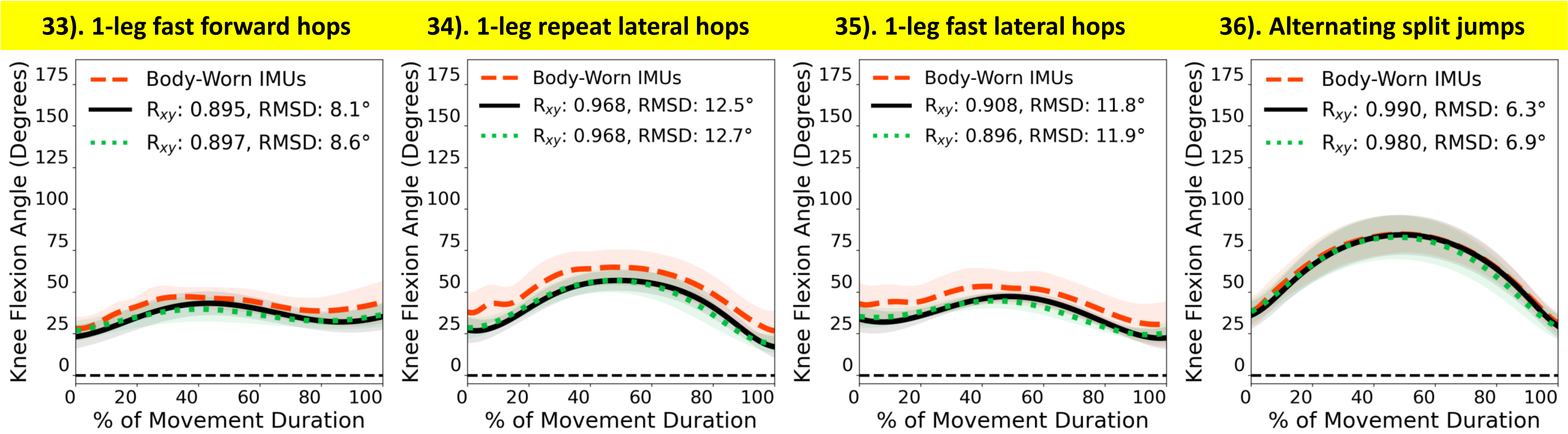
33). 1-leg fast forward hops
34). 1-leg repeat lateral hops
35). 1-leg fast lateral hops
36). Alternating split jumps
Body-Worn IMUs
R_{xy}: 0.895, RMSD: 8.1°
R_{xy}: 0.897, RMSD: 8.6°
Body-Worn IMUs
R_{xy}: 0.968, RMSD: 12.5°
R_{xy}: 0.968, RMSD: 12.7°
Body-Worn IMUs
R_{xy}: 0.908, RMSD: 11.8°
R_{xy}: 0.896, RMSD: 11.9°
Body-Worn IMUs
R_{xy}: 0.990, RMSD: 6.3°
R_{xy}: 0.980, RMSD: 6.9°
Knee Flexion Angle (Degrees)
% of Movement Duration
0
25
50
75
100
125
150
175
0
20
40
60
80
100
