# Supplementary material for: A Simple Minimum-Setup Pipeline for Using Leg-Worn Inertial Sensors to Track Knee Flexion: Validation on 10 Movements
Source: Sensors (Basel). 2026 Jun 10;26(12):3704. doi: 10.3390/s26123704 (PMC13307479; doi:10.3390/s26123704)
Supplement: Supplementary file 1 [file sensors-26-03704-s001.zip › sensors-4323304 _Supplementary Material 2.pdf]

sensors

MDPI

Supplementary Material 2

A Simple Minimum-Setup Pipeline for Using Leg-Worn Inertial Sensors to Track Knee Flexion: Validation on 10 Movements – *Ancillary Data and Sensitivity Analyses*

Ke Song [1,*] and Josh R. Baxter [1]

[1] Department of Orthopaedic Surgery, University of Pennsylvania, Philadelphia, PA 19104, USA
* Correspondence: ke.song@pennmedicine.upenn.edu

Table S1: Descriptive statistics on group-wise trends of intra-subject variability in waveform agreement and magnitude difference

Table S1 below reports group-wise trends (i.e., mean, standard deviation, and inter-quartile range across 10 participants) of intra-subject variability in waveform agreement (R_{xy}) and magnitude difference (RMSD) between IMU and optical motion capture-based knee flexion. The distinction of Table S1 from our main numerical results (Table 1 in the article) is that instead of per-subject average, Table S1 is based on the intra-subject standard deviation of R_{xy} and RMSD, which characterize their variability within each participant (i.e., from movement repetition to repetition, such as step-to-step during walking).

Table S1. Descriptive statistics on group-wise trends of intra-subject variability (i.e., intra-subject standard deviation) in waveform agreement (R_{xy}) and magnitude difference (RMSD) between knee flexion estimated from IMUs vs. motion capture. SD, standard deviation; IQR, inter-quartile range.

**IMU vs. Marker-Based: Intra-Subject Variability of R_{xy} and RMSD in Knee Flexion, n = 10**						
**Movement**	**Pearson correlation coefficient, R_{xy}**			**Root-mean-square difference, RMSD**		
	**Mean**	**SD**	**IQR**	**Mean**	**SD**	**IQR**
Walking	0.007	0.012	0.007	0.9°	0.9°	0.7°
High Step Up	0.001	0.000	0.000	0.8°	0.4°	0.4°
High Step Down *	0.001	0.001	0.001	0.7°	0.6°	0.3°
2-Leg Full Squat	0.000	0.000	0.000	0.3°	0.2°	0.2°
Forward Lunge	0.000	0.000	0.000	0.4°	0.2°	0.2°
Running	0.002	0.001	0.002	1.5°	0.7°	1.1°
Vertical Jump	0.001	0.001	0.001	0.9°	0.5°	0.8°
Forward Jump	0.002	0.002	0.002	1.9°	1.5°	1.8°
Repetitive Jumps	0.010	0.015	0.004	2.4°	1.2°	1.5°
Run-and-Cut	0.013	0.009	0.009	1.7°	0.9°	1.5°
**IMU vs. Markerless Optical: Intra-Subject Variability of R_{xy} and RMSD in Knee Flexion, n = 10**						
**Movement**	**Pearson correlation coefficient, R_{xy}**			**Root-mean-square difference, RMSD**		
	**Mean**	**SD**	**IQR**	**Mean**	**SD**	**IQR**
Walking	0.002	0.001	0.001	0.6°	0.3°	0.2°
High Step Up	0.001	0.001	0.001	0.8°	0.4°	0.5°
High Step Down *	0.001	0.001	0.001	0.6°	0.5°	0.4°
2-Leg Full Squat	0.000	0.000	0.000	0.3°	0.1°	0.2°
Forward Lunge	0.000	0.000	0.000	0.4°	0.3°	0.2°
Running	0.002	0.001	0.001	1.7°	0.8°	1.0°
Vertical Jump	0.001	0.001	0.001	0.7°	0.5°	0.6°
Forward Jump	0.006	0.005	0.006	1.8°	1.3°	1.8°
Repetitive Jumps	0.010	0.009	0.008	2.3°	1.1°	1.6°
Run-and-Cut	0.027	0.018	0.031	2.0°	1.3°	2.3°

* n = 8 due to lost trials caused by technical issues (e.g., dropped Bluetooth streaming signals).

Figure S1: Effect of alternative gain parameter values for IMU fusion algorithm (Madgwick filter) on knee flexion tracking

We performed a sensitivity analysis on the effect of Madgwick filter gain β on tracking results, using walking, squats, and running trials from one participant (10 seconds of data from each trial). Results showed that altering β from 0.1 (relying more on gyroscope) to 1.0 (relying more on accelerometer) had little effect on estimated knee flexion for any movement, with RMSD ≤ 1.2° compared to results based on default gain (β = 0.5) for walking and squats and RMSD ≤ 2.7° for running (Figure S1).

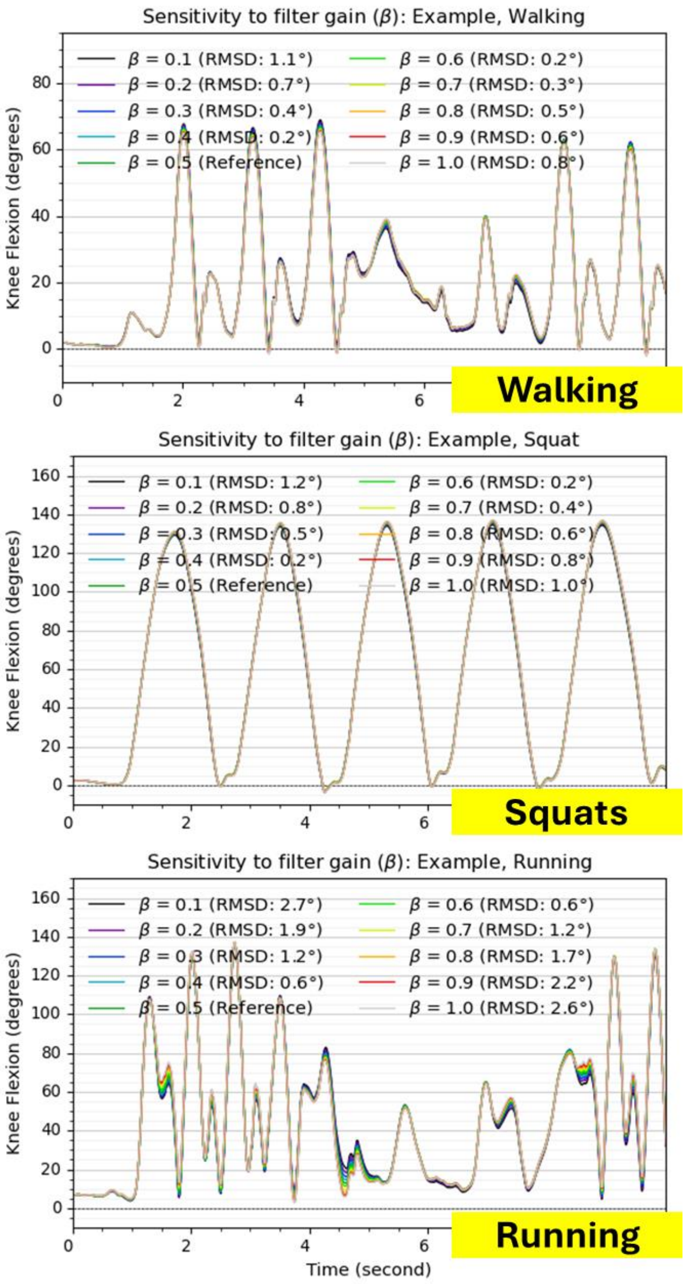

Figure S1. Comparing IMU knee flexion tracking results with various Madgwick filter gains β from 0.1 to 1. A lower gain represents more reliance on gyroscope data for fusion, whereas a higher gain represents more reliance on accelerometer data. Altering the gain had little effect on estimated knee flexion: compared to results based on default (β = 0.5), when using a different gain, RMSD over the 10 seconds of data shown was ≤ 1.2° for walking and squats, and ≤ 2.7° for running. (In these walking and running trials, this participant turned around at 4 – 8 seconds before resuming the movement.)

Figure S2: Effect of prepending 5 seconds of data "freeze" for initializing the IMU fusion algorithm on knee flexion tracking

To verify the effect of using the "freeze" (static data padding) on short versus longer trials, we performed a sensitivity analysis on the walking, squats, and running trials from one participant, each using the first 10 seconds of data. Results showed that not adding the 5-second "freeze" resulted in irregular IMU tracking artifacts during the initial seconds of "real" data, especially in the faster running trial (Figure S2). Data after the initial seconds were largely identical regardless of whether the "freeze" procedure was on or off.

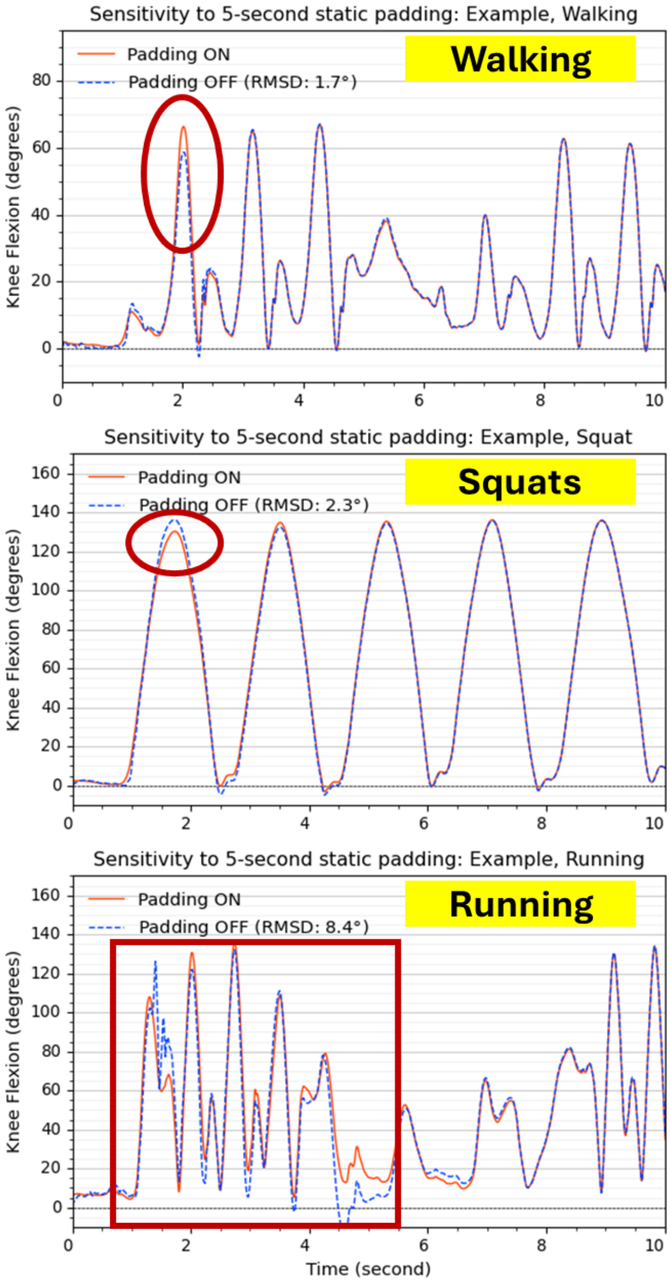

Figure S2. Comparing IMU knee flexion tracking results with the 5-second "freeze" procedure (static data padding) used (default) versus unused. Not adding the 5-second "freeze" resulted in irregular IMU tracking artifacts during the initial seconds of data, especially in the faster running trial (dark red square/circles). Data after the initial seconds were largely identical regardless of the "freeze". RMSD values represent the average magnitude difference between the two waveforms over the 10 seconds of data shown.

Figure S3: Effect of alternative calibration trials for knee flexion axis alignment (principal component analysis) on knee flexion tracking

We performed a sensitivity analysis on the principal component analysis (PCA) trial of choice where we used two alternative sagittal motion trials (2-leg full squats or first 5 seconds of running) for PCA-based alignment and compared results from one participant. Despite switching to different PCA trials, IMU-estimated knee flexion remained highly similar to walking-based PCA alignment, with RMSD ≤ 1.6° for the walking trial, 0.8° for the squats trial, and ≤ 2.6° for the running trial. Notably, using a specific trial for PCA alignment did not favor the tracking results for that same trial over other trials.

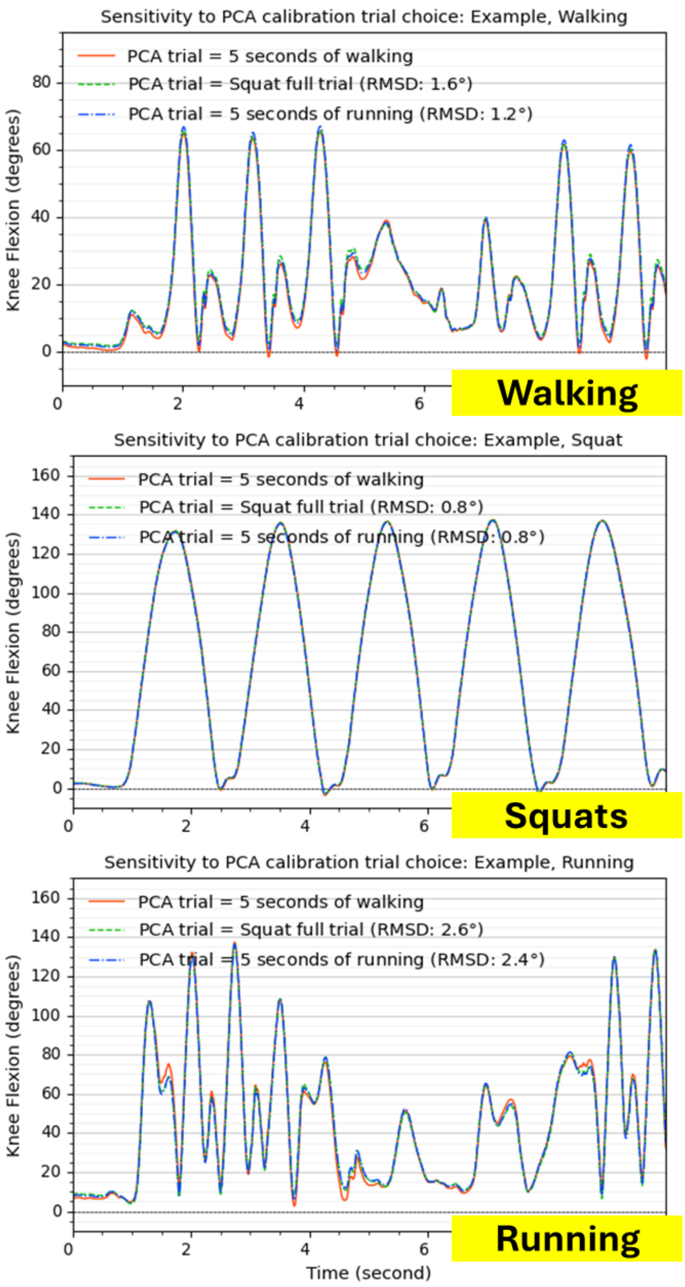

Figure S3. Comparing IMU knee flexion tracking results with alternative trials for PCA-based knee flexion axis alignment: 5 seconds of walking (default), full squatting trial, or 5 seconds of running. As all three trials featured primarily sagittal knee motion, using alternative trials for PCA produced highly similar results to walking-based (RMSD ≤ 2.6° over the 10 seconds of data shown). Notably, using a specific trial for PCA did not favor the tracking results for that same trial over other trials.
